# Antibody Responses to NY-ESO-1 in Primary Breast Cancer Identify a Subtype Target for Immunotherapy

**DOI:** 10.1371/journal.pone.0021129

**Published:** 2011-06-17

**Authors:** Ahmed Hamaï, Karine Duperrier-Amouriaux, Pascale Pignon, Isabelle Raimbaud, Lorenzo Memeo, Cristina Colarossi, Vincenzo Canzonieri, Tiziana Perin, Jean-Marc Classe, Mario Campone, Pascal Jézéquel, Loïc Campion, Maha Ayyoub, Danila Valmori

**Affiliations:** 1 Institut National de la Santé et de la Recherche Médicale, Unité 892, CLCC René Gauducheau, Saint Herblain, France; 2 Pathology Unit, Mediterranean Institute of Oncology, Catania, Italy; 3 Pathology, Centro di Riferimento Oncologico, IRCCS, National Cancer Insitute, Aviano, Pordenone, Italy; 4 Department of Surgery, CLCC René Gauducheau, Saint Herblain, France; 5 Department of Medical Oncology, CLCC René Gauducheau, Saint Herblain, France; 6 Department of Onco-biology, CLCC René Gauducheau, Saint Herblain, France; 7 Department of Biostatistics, CLCC René Gauducheau, Saint Herblain, France; 8 Institut National de la Santé et de la Recherche Médicale, Unité 892, IRT UN, Nantes, France; 9 Faculty of Medicine, University of Nantes, Nantes, France; University of Hong Kong, Hong Kong

## Abstract

The highly immunogenic human tumor antigen NY-ESO-1 (ESO) is a target of choice for anti-cancer immune therapy. In this study, we assessed spontaneous antibody (Ab) responses to ESO in a large cohort of patients with primary breast cancer (BC) and addressed the correlation between the presence of anti-ESO Ab, the expression of ESO in the tumors and their characteristics. We found detectable Ab responses to ESO in 1% of the patients. Tumors from patients with circulating Ab to ESO exhibited common characteristics, being mainly hormone receptor (HR)^−^ invasive ductal carcinomas of high grade, including both HER2^−^ and HER2^+^ tumors. In line with these results, we detected ESO expression in 20% of primary HR^−^ BC, including both ESO Ab^+^ and Ab^−^ patients, but not in HR^+^ BC. Interestingly, whereas expression levels in ESO^+^ BC were not significantly different between ESO Ab^+^ and Ab^−^ patients, the former had, in average, significantly higher numbers of tumor-infiltrated lymph nodes, indicating that lymph node invasion may be required for the development of spontaneous anti-tumor immune responses. Thus, the presence of ESO Ab identifies a tumor subtype of HR^−^ (HER2^−^ or HER2^+^) primary BC with frequent ESO expression and, together with the assessment of antigen expression in the tumor, may be instrumental for the selection of patients for whom ESO-based immunotherapy may complement standard therapy.

## Introduction

Human breast cancers (BC) are highly heterogeneous with respect to their biologic and clinical profiles. Currently, clinical management relies on known prognostic factors, including hormone receptor (HR) and HER2 status. Evaluation of the expression of these factors is valuable for predicting responsiveness to therapies that specifically target them [1], [2], [3]. Despite treatment, however, a number of patients progress and in patients who do not express these markers, therapeutic options remain limited.

Cancer-testis (CT) antigens are encoded by a group of genes expressed in human germ line cells, silenced in somatic cells in normal adult tissues, and aberrantly re-expressed in cancerous cells in tumors of different histological types [4]. About half of the known genes encoding CT antigens are located in the X-chromosome (CT-X), and together represent approximately 10% of all genes in the X [5]. Because of their highly tumor-specific expression, CT antigens are excellent targets of anti-tumor immune responses. For several group members, specific immune responses can develop spontaneously in some cancer patients bearing antigen-expressing tumors. One of the most studied family members, NY-ESO-1 (ESO), is remarkably immunogenic, eliciting spontaneous antibody (Ab) and T cell responses [6], [7]. Because of its high immunogenicity, ESO has been selected among the high priority candidates for the development of generic anti-cancer vaccines [8]. The development is promising, with some formulations showing high immunogenicity in cancer patients [9]. Clinical efficacy of ESO-based vaccination, however, remains to be demonstrated, through upcoming clinical trials targeting specific patient populations. Other approaches targeting ESO-expressing tumors through adoptive transfer therapy are also being developed and have recently yielded the first evidence of clinical efficacy [10]. Because of the potential of ESO for immunotherapy, identification of BC patients with spontaneous immune responses to the antigen could be instrumental for the selection of the patients who could benefit from ESO-based immunotherapy. Here we show that the presence of ESO Ab identifies a subtype of primary BC with frequent ESO expression and, together with the assessment of antigen expression in the tumor, is instrumental for the selection of patients for whom ESO-based immunotherapy may complement standard therapy.

## Results

### Assessment of ESO-specific Ab in patients with primary BC

**Figure 1:**
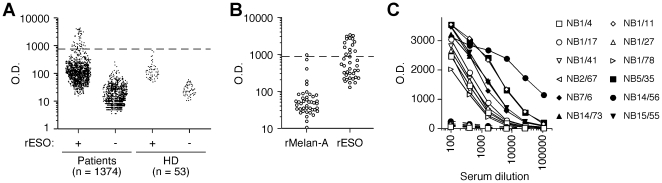
Assessment of circulating ESO-specific Ab in patients with primary BC. **A**. Sera from BC patients and control healthy donors (HD) were screened by ELISA at a serum dilution of 1∶100 on rESO-coated plates and on control plates with no antigen. Samples were considered positive when the optical density (OD) value obtained on rESO-coated plates was higher than the mean+6xSD of OD values obtained with sera from HD on rESO-coated plates. **B**. Samples that scored positive in **A** were assessed at a dilution of 1∶400 on plates coated with rESO or rMelan-A. Samples were confirmed as ESO Ab^+^ if the OD value obtained on rESO-coated plates was both higher than the mean+6xSD of OD values obtained on rMelan-A-coated plates for all patients and 3 folds higher than the OD value obtained for the same serum on rMelan-A-coated plates. **C**. Serial dilutions of ESO Ab^+^ sera were assessed on rESO (solid lines) and rMelan-A (dotted lines) coated plates and serum titer was calculated as the serum dilution yielding 50% of maximal OD on rESO-coated plates.

We assessed the presence of circulating ESO-specific Ab in a cohort of 1374 patients with primary BC seen at our institution from 1999 to 2008. ESO Ab were assessed by ELISA in sera taken at the time of first surgery, using a recombinant ESO protein (rESO) produced by expression in E. coli, as described previously [11]. Samples from patients and control healthy donors were screened on rESO-coated microplates and on control plates with no antigen. Data obtained are shown in Figure 1A. Samples that scored as positive in this first screening were further assessed with an unrelated control protein similarly produced in E. coli, to eliminate sera that non-specifically reacted with bacterial contaminants in the preparation (Figure 1B). Following this analysis, 12 patients (1%) were confirmed as positive for serological IgG responses to ESO (Ab^+^) and were further assessed in a titration assay to determine antibody titers (Figure 1C). The latter varied from 1∶150 to 1∶22000 (average 1∶2800).

### ESO expression in primary BC tumors from ESO Ab^+^ patients and correlation with HR and HER2 status

**Figure 2:**
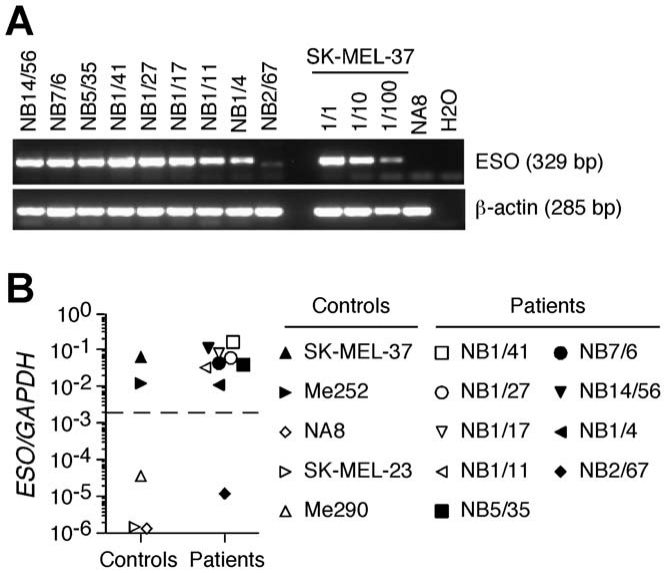
Assessment of ESO expression in BC tumors from ESO Ab^+^ patients. ESO expression in cryopreserved BC tumors from ESO Ab^+^ patients was assessed by semi-quantitative PCR (**A**) and qPCR (**B**) using specific primers and ESO^+^ (SK-MEL-37, Me252) and ESO^−^ (NA8, SK-MEL-23, Me290) tumor cell lines as internal controls.

The characteristics of the ESO Ab^+^ patients and of the corresponding tumors are reported in Table 1 (upper part). For 9 of them cryopreserved tumor tissue was available. We assessed ESO expression in these samples by semi-quantitative PCR using previously validated primers (Figure 2A). ESO expression was further confirmed and quantified by qPCR (Figure 2B). Tumor lines and healthy tissues were used as internal controls (Figure 2 and Supporting Figure S1). In support of the results of the serological analysis, we found ESO expression in 8 of the 9 tumors. Tumors from ESO Ab^+^ patients were mostly invasive ductal carcinomas (IDC), 1 was diagnosed as medullary breast cancer (MBC) and 2 as lymph node metastases with unknown primary. Tumors were of high grade, estrogen receptor (ER)^−^ and progesterone receptor (PR)^−^. Three of them over-expressed HER2. Five of the patients had a family history of BC, 4 of them had other cancers and 2 of them had deceased at the time of analysis. It is noteworthy that the only patient for whom we could not detect ESO expression in the breast tumor (that at variance with the others was low grade, ER^+^) was diagnosed with malignant melanoma, that frequently expresses ESO, a few months after the diagnosis of BC.

**Table 1:** BC patients with detectable ESO Ab and/or ESO expression and characteristics of the corresponding tumors.

Patient	ESO Ab	ESO exp*	ER†	PR†	HER2‡	Age§	SBR∥	Histological type	Other tumors	Survival§§	Relapse	Family history∫∫
NB1/78	+	na	−	−	−	26	III	IDC††	-	D	Liver	+
NB1/11	+	+	−	−	+	61	III	IDC	Meningioma	A	-	+
NB5/35	+	+	−	−	nd	52	up**	up	-	A	-	−
NB1/27	+	+	−	−	−	59	III	IDC	-	A	-	−
NB15/55	+	na	−	−	−	48	up	up	Cervical	A	-	+
NB1/41	+	+	−	−	−	61	III	IDC	-	A	Skin, LN	+
NB1/4	+	+	−	−	−	57	III	IDC	-	A	-	−
NB14/73	+	na	−	−	+	68	III	IDC	-	A	-	−
NB1/17	+	+	−	−	−	37	III	IDC	-	D	LN	+
NB7/6	+	+	−	−	+	65	III	IDC	Spinocellular	A	-	−
NB14/56	+	+	−	−	−	66	III	MBC‡‡	-	A	-	−
NB2/67	+	−	+	−	−	71	I	IDC	Melanoma	A	-	−
NB1/8	−	+	−	−	−	65	II	IDC	-	A	-	+
NB1/13	−	+	−	−	−	48	III	IDC	-	A	-	−
NB1/15	−	+	−	−	−	56	II	IDC	-	D	Pleural, skin, LN	+
NB1/26	−	+	−	−	−	65	III	IDC	-	A	-	−
NB1/37	−	+	−	−	+	83	III	ILC∫	-	A	-	−
NB1/38	−	+	−	−	−	55	III	Basal-like	-	A	-	−
NB1/47	−	+	−	−	−	58	III	IDC	-	A	-	−
NB1/80	−	+	−	−	−	74	III	IDC	-	D	Skin	−
NB1/85	−	+	−	−	−	42	III	MBC	-	A	-	+
NB14/78	−	+	−	−	−	62	III	IDC	-	A	-	−

* ESO mRNA expression in frozen tumor specimens was assessed by RT-PCR. na, not available.

† ER and PR expression was assessed by IHC staining of paraffin-embedded tumor specimens.

‡ HER2 was assessed by IHC staining in paraffin-embedded tumor specimens and gene amplification was confirmed by FISH analysis. nd, not done.

§ Age at diagnosis.

∥ SBR, Scarff-Bloom-Richardson tumor grade.

** Lymph node metastasis with unknown primary (up).

†† IDC, invasive ductal carcinoma.

‡‡ MBC, medullary breast cancer.

∫ ILC, invasive lobular carcinoma.

§§ D, dead; A, alive.

∫∫ Known family history of breast cancer.

### Expression of ESO in ER^−^ and ER^+^ BC

**Figure 3:**
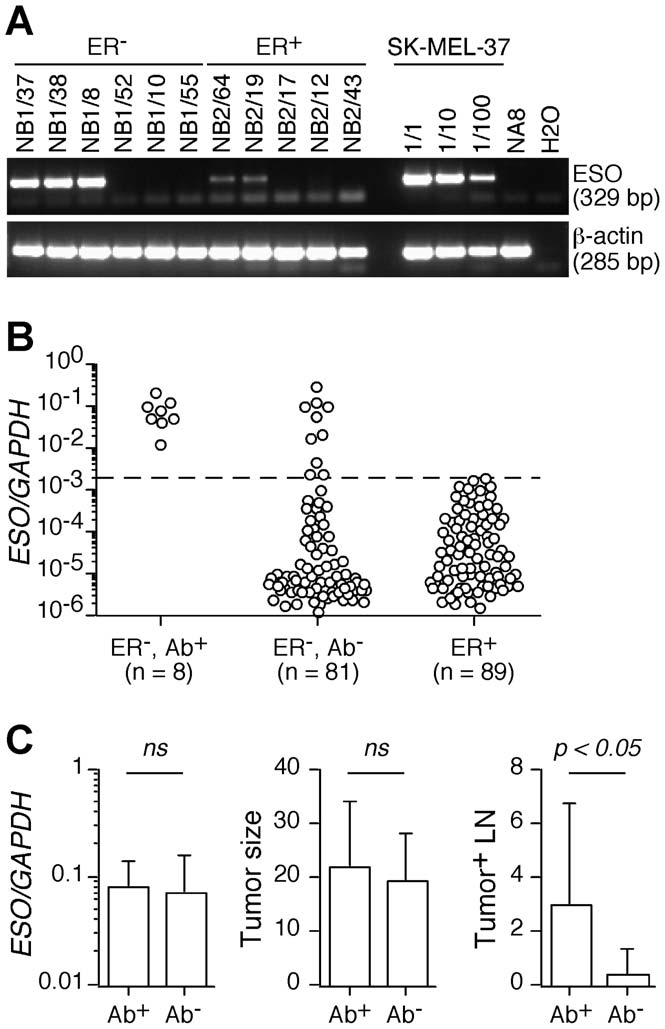
Assessment of ESO expression in ER^−^ and ER^+^ BC tumors. **A** and **B**. ESO expression in cryopreserved ER^−^ and ER^+^ BC tumors was assessed by semi-quantitative PCR (**A**) and qPCR (**B**). **C**. The level of ESO expression in the tumor as assessed by qPCR (left panel, mean ± SD), the size of the primary tumor at diagnosis (middle panel, mean ± SD) and the number of tumor-invaded lymph nodes (right panel, mean ± SD) are shown for ESO Ab^+^ (n = 8) and ESO Ab^−^ (n = 10) patients with ESO-expressing tumors. Statistical analyses were performed using a two-tailed t-test.

The correlation between ESO expression and the ER status found for ESO Ab^+^ patients prompted us to extend the assessment of ESO expression to a larger group of ER^−^ tumors and a similar group of ER^+^ tumors from patients in the cohort. Samples were assessed by semi-quantitative PCR and qPCR (Figure 3A and 3B). We found significant ESO expression in 18 (20%) of ER^−^ BC including the 8 patients previously identified through serological analysis and 10 additional patients without detectable ESO Ab. In contrast, we did not find significant levels of ESO expression in the ER^+^ tumors. The characteristics of ESO Ab^−^ patients with detectable ESO expression and of the corresponding tumors are reported in Table 1 (lower part). Similar to the tumors from ESO Ab^+^ patients, the majority of the tumors from these patients were high grade and PR^−^. The large majority was IDC, with 1 diagnosed as MBC and 1 as basal-like cancer (BLC). One of the tumors over-expressed HER2. Three of the patients had a family history of breast cancer, none of them had other tumors and 2 of them had deceased at the time of analysis. Thus, ESO-expressing Ab^−^ patients had characteristics similar to those of the ESO Ab^+^ group. It is noteworthy that the expression levels of ESO in tumors from ESO Ab^+^ patients were not significantly different from those found in ESO-expressing tumors from ESO Ab^−^ patients (Figure 3C). We found no significant difference in tumor size between the 2 groups. Interestingly, however, the number of tumor-invaded lymph nodes was, in average, significantly higher in Ab^+^ patients than in Ab^−^ patients (Figure 3C).

**Figure 4:**
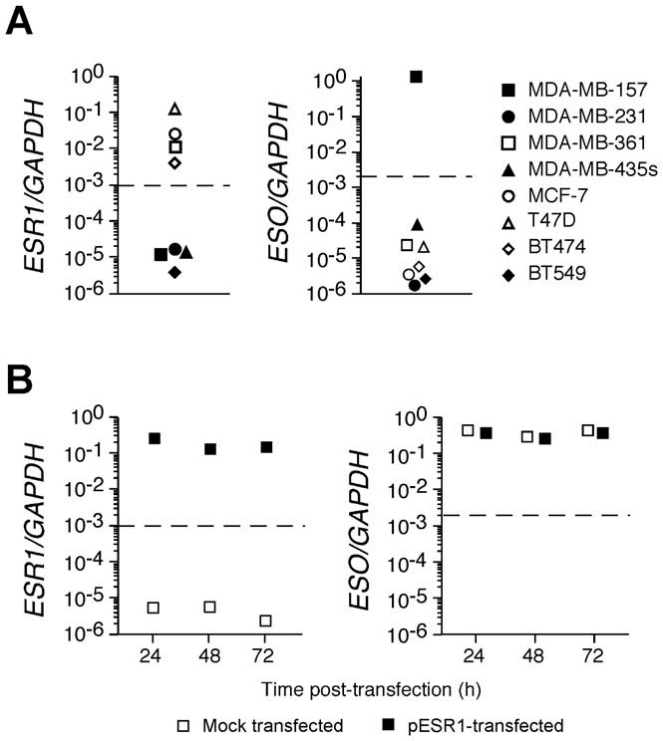
Assessment of ESO expression in BC lines. **A**. ER (*ESR1*) and ESO expression in the indicated BC lines was assessed by qPCR. **B**. MDA-MB-157 cells were transfected with a plasmid encoding the full length ER (pESR1) or were mock transfected and ER and ESO expression was assessed 24, 48 and 72 h post-transfection by qPCR.

To further address the relationship between the ER status of BC tumors and ESO expression, we assessed ESO expression in 8 BC tumor lines including 4 ER^−^ and 4 ER^+^ lines [12]. As shown in Figure 4A, we found ESO expression in one of the ER^−^ lines, MDA-MB-157, but in none of the ER^+^ lines. To address if expression of ER directly affects ESO expression in BC tumors, we transfected MDA-MB-157 cells with an ER-encoding plasmid (pESR1). ER expression was indeed efficiently induced in MDA-MB-157 cells after transfection but no significant effect on ESO expression was observed (Figure 4B).

### Expression of ESO in triple negative breast cancers

**Figure 5:**
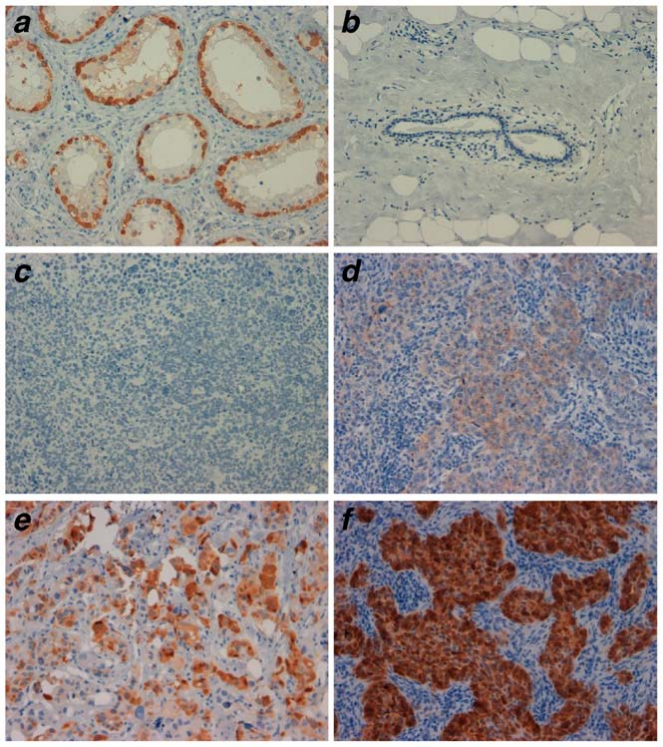
Expression of ESO in triple negative BC. Expression was assessed by IHC staining of paraffin-embedded tumors using the ESO-specific mAb E978. Tumors were scored based on the percentage of positive cells (-, 0-rare; 1, <10%; 2, 10–25%; 3, 25–50%; 4, >50%) and the staining intensity (A, faint; B, moderate; C, strong). Testis (positive control, **a**), normal breast (negative control, **b**) and examples of triple negative tumors scored as negative (**c**), 4B (**d**), 3C **(e**) and 4C (**f**) are shown. Magnification: ×20.

Patients with ER^−^/PR^−^/HER2^−^ BC tumors (called triple negative, TN) have poor prognosis and limited therapeutic options. To further validate the ESO expression data obtained in the first cohort, we assessed a separate cohort of 42 TN high-grade IDC BC tumors collected from 1999 to 2003 at the Italian National Cancer Institute (Aviano, Italy). For these tumors, paraffin-embedded blocks were available for analysis and were assessed for expression of ESO protein by IHC. Examples of the results obtained are shown in Figure 5 and data obtained for all tumors are summarized in Supporting Table S1. We found detectable ESO expression in 10 (24%) of the samples. Both the proportion of BC cells expressing ESO and the intensity of staining were variable among samples, in line with previous findings in tumors of different histological types [13], [14]. TN BC encompass BLC, that are believed to originate from basal stem precursors [15]. To explore a possible correlation between ESO expression in TN tumors and BLC, we assessed the expression of several basal-like associated markers, including epithelial growth factor receptor (EGFR), p63, cytokeratine (CK)5 and CK14 (Supporting Table S1). As expected, we found a frequent expression of these markers in TN tumors. We failed, however, to find a correlation between ESO expression and any of the markers (Supporting Figure S2). Thus expression of ESO, although frequent in TN tumors, is not restricted to the basal-like type, in agreement with the presence of HER2^+^ tumors, that are distinct from basal-like tumors, among ESO-expressing cases (Table 1).

## Discussion

Clinical management of primary BC mainly rests on the assessment of HR and HER2 expression along with tumor grade. Patients bearing HR-expressing tumors are eligible, in addition to chemotherapy, for hormonotherapy, and those with HER2 over-expressing tumors to treatment with herceptin (trastuzumab). With the aim of developing improved approaches for the treatment of breast cancer, in recent years, molecular classes of BC have been distinguished based on their gene-expression profiles. The main classification [15] proposes five classes: normal-like; Luminal-A, mostly ER^+^, low grade; Luminal-B, mostly ER^+/low^, high grade; BLC, mostly ER^−^, PR^−^ and HER2^−^ (TN); and HER2^+^ BC. The molecular types also correlate with prognosis and response to therapy. Namely, whereas Luminal-A tumors are generally indolent and sensitive to hormonotherapy, Luminal-B tumors and ER^+^ HER2^+^ tumors are less responsive. Both HER2^+^ tumors and BLC are aggressive, with HER2^+^ tumors being selectively sensitive to trastuzumab and both HER2^+^ tumors and BLC possibly more sensitive to preoperative chemotherapy [16]. Although the immediate added clinical value of the molecular classification is limited by its correlation with HR, HER2 status and tumor grade, the demonstration of differential gene expression profiles in these tumor types is providing a broader view of their phenotype that could reveal new type-specific therapeutic targets.

The clinical efficacy of trastuzumab in patients with HER2 over-expressing tumors has clearly demonstrated the possibility to impact on disease progression through immunotherapy and encourages the development of anti-cancer vaccines in breast cancer, provided that appropriate target tumor antigens and patient groups are identified. In this study, we have assessed spontaneous Ab responses to one of the most immunogenic CT antigens, ESO, in a large cohort of patients with primary BC. This is, in our knowledge, the first systematic assessment of spontaneous immune responses to a member of the CT antigens group in primary BC. The results obtained provide important cues for ESO-based immunotherapy in these patients. We identified a small group of patients (1%) with detectable spontaneous Ab responses to ESO. Tumors from patients with circulating Ab to ESO exhibited common characteristics, being mainly HR^−^ IDC of high grade, including HER2^−^ and HER2^+^ tumors. In line with these results, we detected ESO expression in 20% of primary HR^−^ BC from both ESO Ab^+^ and Ab^−^ patients, but not in HR^+^ BC. Expression levels in ESO^+^ BC were not significantly different between ESO Ab^+^ and Ab^−^ patients, but the former had, in average, significantly higher numbers of tumor-infiltrated lymph nodes. Thus, the presence of ESO Ab identifies a subtype of HR^−^ primary BC (HER2^+^ or HER2^−^) with frequent ESO expression.

The molecular basis of the relationship between the HR status of BC tumors and the expression of ESO is unknown. By transfecting an ER^−^ ESO^+^ BC line with an ER-encoding plasmid we failed to detect any significant alteration of ESO expression, suggesting that the correlation between the HR status of BC and the expression of ESO may reflect a particular molecular subtype of the tumor rather than a direct effect of HR on ESO expression.

We confirmed the frequent expression of ESO at the protein level by IHC in a separate cohort of TN tumors. Interestingly, TN BLC, that are believed to originate from basal stem precursors, are characterized by frequent dysfunction of the BRCA1 pathway, which is important in DNA repair, activation of cell cycle checkpoints and maintenance of chromosomal stability, including X chromosome inactivation (Xi) [17], [18]. It is therefore tempting to propose the existence of a possible link between CT-X expression in BC and loss of Xi. These considerations, however, are complicated by our finding that CT-X expression is not limited to the TN BLC group, as a fraction of CT-X expressing HR^−^ tumors in this study were positive for HER2, whose over-expression identifies a subgroup of BC with molecular characteristics distinct from BLC [15].

The finding that patients with primary BC bearing sometimes relatively small ESO-expressing tumors can mount significant serological responses to ESO is somewhat unexpected, with respect to previous studies that have reported serological responses to ESO in patients bearing significant tumor load, that in some instances become ESO Ab^−^ after removal of the tumor [19]. These results could imply a particularly high immunogenicity of BC or/and that the primary lesion may not represent the actual total tumor mass present at diagnosis in these patients. Interestingly however, we found that the presence of ESO Ab in patients with ESO-expressing BC correlates with the presence of the tumor in the local lymph nodes, suggesting that the latter may be required for the development of spontaneous anti-tumor response. Another important implication of our findings is that ESO may represent a highly specific marker for the detection of tumor cells outside the primary tumor site, i.e. in the circulation, lymph nodes or bone marrow.

In conclusion, the results of our study indicate that the presence of circulating antibodies to ESO identifies a group of HR^−^ BC with frequent ESO expression and, together with the assessment of antigen expression in the tumor, may be instrumental for the selection of patients who may benefit from recently developed highly immunogenic ESO-based vaccines [9]. Anti-cancer vaccination as a complement for standard therapy is particularly attractive in this group of patients, including those bearing TN tumors, for whom therapeutic options are limited, and for patients with HER2^+^ER^−^ tumors where it may be combined with trastuzumab.

## Materials and Methods

### Patients, sera, tumors, normal tissues and tumor cell lines

Sera and surgical tumor specimens from breast cancer patients seen at CLCC René Gauducheau (Nantes-Saint Herblain, France) at the time of first surgery, as well as sera from healthy donors (HD), were collected, upon written informed consent and approval by the Comité de Protection des Personnes Ouest IV – Nantes, France, and cryopreserved. Specimens of triple negative tumors were obtained at the Italian National Cancer Institute (Aviano, Italy), upon written informed consent and approval by the Ethics Committee of the Centro di Riferimento Oncologico, Aviano, Italy, and were fixed in formalin and embedded in paraffin. Total RNA from human normal tissues was purchased from Ambion Inc. Tumor cell lines were maintained in culture in complete DMEM medium (Gibco, Invitrogen, Cergy Pontoise, France) supplemented with 10% fetal calf serum.

### Detection of circulating ESO-specific antibodies

Antibody (Ab) responses to ESO were assessed by ELISA, as previously described [9], [11], using recombinant ESO protein (rESO) or control recombinant Melan-A protein (rMelan-A) produced in E. coli [20]. Briefly, rESO-, rMelan-A- or un-coated plates were incubated with the indicated dilutions of patients or HD sera, washed, incubated with alkaline phosphatase-coupled goat anti-human IgG secondary Ab (Southern Biotechnology, CliniSciences SA, Montrouge, France) and the reaction was revealed using the Attophose substrate (Promega, Charbonnières-les-Bains, France).

### Assessment of ESO expression by semi-quantitative and quantitative PCR

Total RNA was prepared from frozen tumor specimens and from tumor cell lines using the NucleoSpin® RNA II kit (Macherey-Nagel, Hoerdt, France). Where indicated, MDA-MB-157 cells (1×10^6^) were transfected with 1 µg of a recombinant pCMV6-XL5 plasmid encoding the full length ER (pESR1, OriGene, CliniSciences SA, Montrouge, France) or mock transfected using the Cell Line Nucleofector kit V (Amaxa, Köln, Germany) and a Nucleofector II electroporation device (Amaxa), according to the manufacturer's instructions, and total RNA was extracted 24, 48 and 72 h following transfection. cDNA synthesis was performed using Promega Reverse Transcription System A3500 (Promega). Semi-quantitative PCR was performed using GoTaq® Flexi DNA Polymerase (Promega). cDNA integrity was tested by amplification of β-actin mRNA and ESO mRNA expression was assessed using the following primers: forward primer: 5′-ATGGATGCTGCAGATGCGG-3′ and reverse primer: 5′-GGCTTAGCGCCTCTGCCCTG-3′. Quantitative PCR (qPCR) was performed with a TaqMan assay and an ABI 7000 system (Applied Biosystems, Courtaboeuf, France) using assays-on-Demand Gene Expression probes for *ESO* (Hs00265824_m1, Applied Biosystems) and *ESR1* (Hs00174860_m1, Applied Biosystems) and previously described *GAPDH*-specific Taqman probe and primers [21]. Relative mRNA expression was calculated as 2^(Ct GAPDH - Ct Test gene)^.

### Assessment of ESO and basal-like markers expression in tumor samples by IHC

5-µm cuts of standard formalin-fixed, paraffin-embedded tissue specimens were applied to IHC slides, heated at 60°C and deparaffinized. Following antigen retrieval, samples were stained using mAb specific for ESO (E978, Invitrogen, Milano, Italy), CK5 (XM26, Novocastra, Menarini Florence, Italy), CK14 (LL002, Ventana Medical Systems, Tucson, Arizona), EGFR (3C6, Ventana Medical Systems) and p63 (4A4, Dako, Milano, Italy) and revealed with the Envision Plus system with DAB (Dako). Counterstaining was performed with a hematoxylin solution. Tumors were scored based on the percentage of positive cells (-, 0-rare; 1, <10%; 2, 10–25%; 3, 25–50%; 4, >50%) and the staining intensity (A, faint; B, moderate; C, strong).

## Supporting Information

Figure S1 **Assessment of ESO expression in normal tissues.** ESO expression was assessed by semi-quantitative PCR (**A**) and qPCR (**B**) following reverse transcription of mRNA from a panel of normal tissues and was used to determine the cut-off between positive and negative tumor samples.
(TIF)

Figure S2 **Expression of ESO and of basal-like carcinoma associated markers in triple negative BC.** Expression of ESO, CK5, CK14, EGFR and p63 was assessed by IHC staining of paraffin-embedded tumors from a cohort of 42 patients with triple negative BC (Supporting Table S1). The percentage of tumors expressing ESO within the entire cohort and of tumors expressing the indicated basal-like associated markers within the entire cohort or among ESO^+^ tumors are shown. na, non applicable.
(TIF)

Table S1 **Expression of ESO and of basal-like carcinoma associated markers in a cohort of triple negative breast cancers.**
(DOC)
