# Supplementary figures and images for: Antibody Responses to NY-ESO-1 in Primary Breast Cancer Identify a Subtype Target for Immunotherapy
Source: PLoS One. 2011 Jun 17;6(6):e21129. doi: 10.1371/journal.pone.0021129 (PMC3117860; doi:10.1371/journal.pone.0021129)

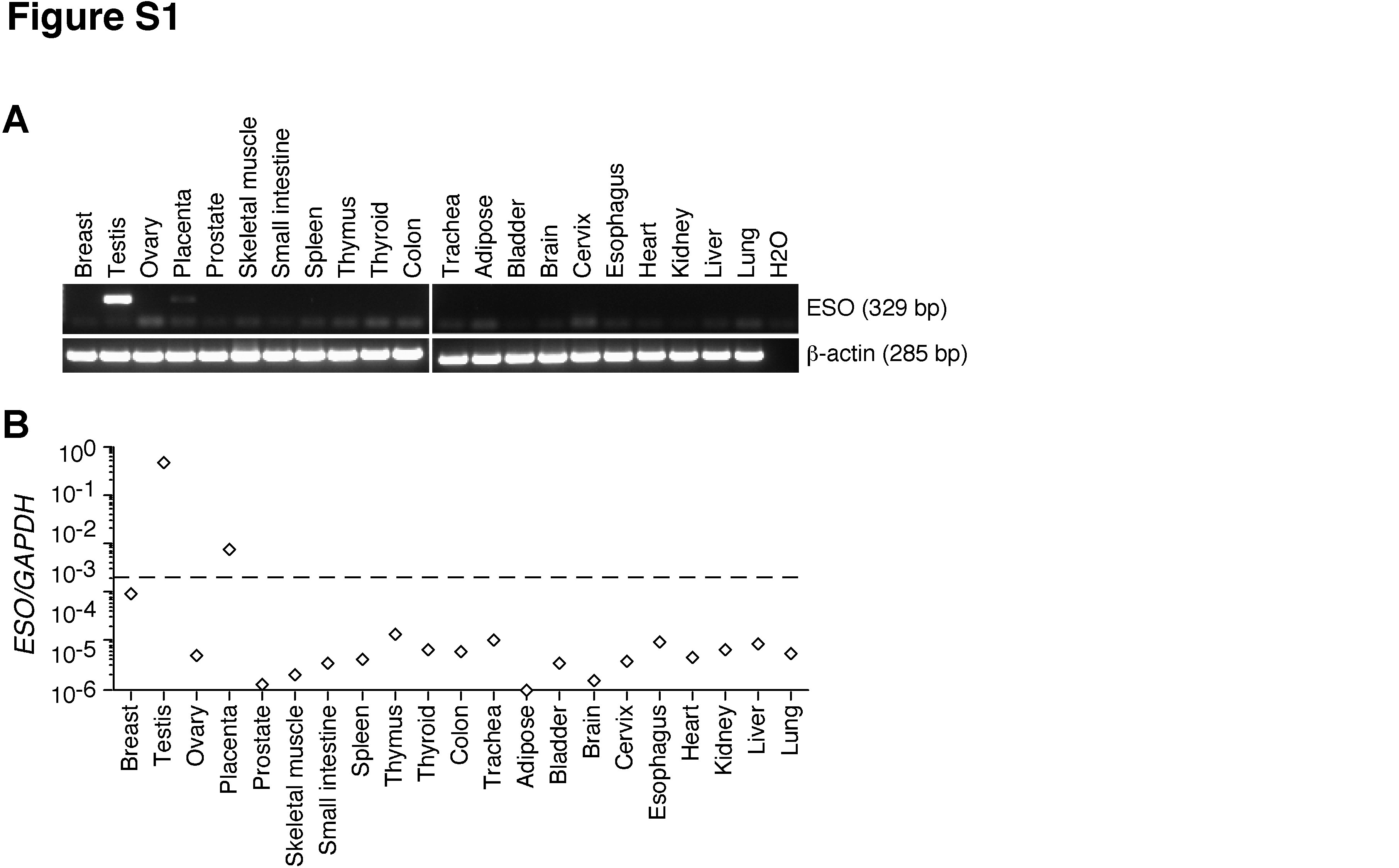

Supplement: Figure S1 — Assessment of ESO expression in normal tissues. ESO expression was assessed by semi-quantitative PCR (A) and qPCR (B) following reverse transcription of mRNA from a panel of normal tissues and was used to determine the cut-off between positive and negative tumor samples. (TIF) [file pone.0021129.s001.tif]

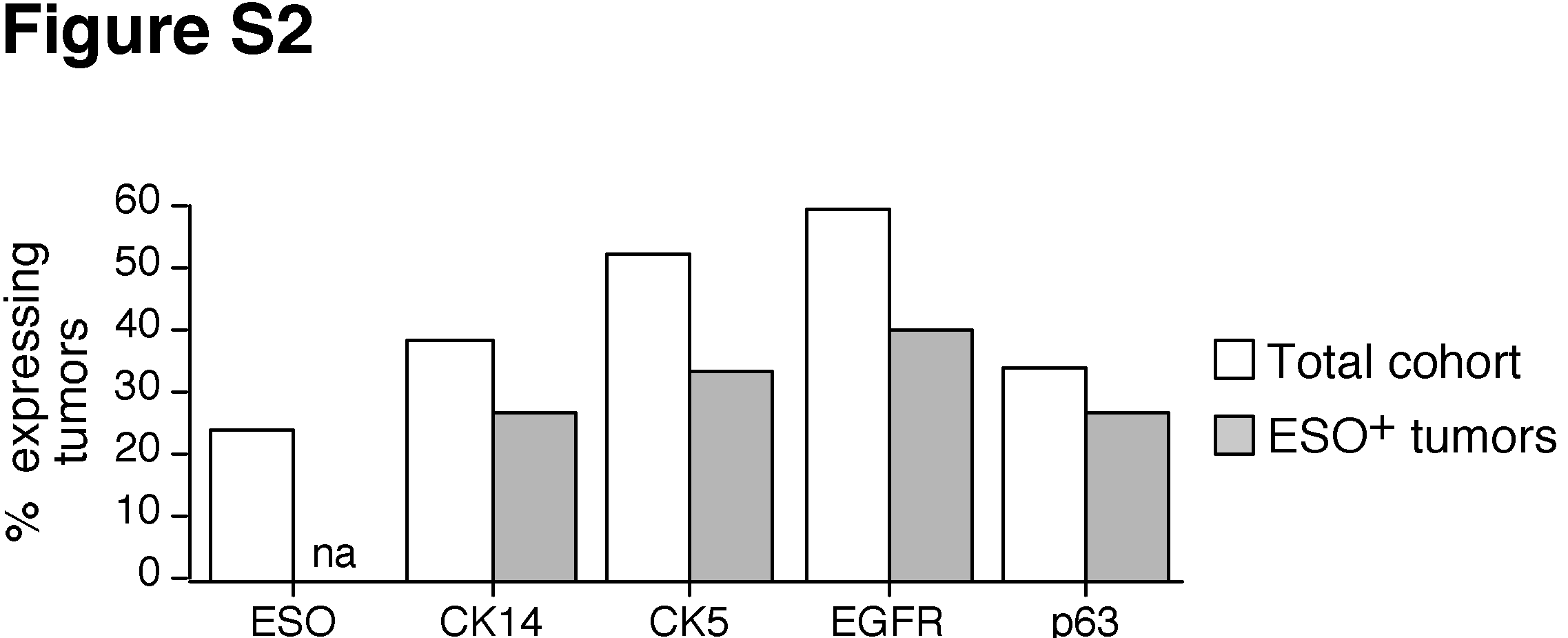

Supplement: Figure S2 — Expression of ESO and of basal-like carcinoma associated markers in triple negative BC. Expression of ESO, CK5, CK14, EGFR and p63 was assessed by IHC staining of paraffin-embedded tumors from a cohort of 42 patients with triple negative BC (Supporting Table S1). The percentage of tumors expressing ESO within the entire cohort and of tumors expressing the indicated basal-like associated markers within the entire cohort or among ESO+ tumors are shown. na, non applicable. (TIF) [file pone.0021129.s002.tif]
