# Supplementary material for: Antibody Responses to NY-ESO-1 in Primary Breast Cancer Identify a Subtype Target for Immunotherapy
Source: PLoS One. 2011 Jun 17;6(6):e21129. doi: 10.1371/journal.pone.0021129 (PMC3117860; doi:10.1371/journal.pone.0021129)
Supplement: Table S1 — Expression of ESO and of basal-like carcinoma associated markers in a cohort of triple negative breast cancers. (DOC) [file pone.0021129.s003.doc]

## Table S1. Expression of ESO and of basal-like carcinoma associated markers in a cohort of triple negative breast cancers.

| **Patient** | **Age**^*^ | **ESO**^†^ | **CK14**^†^ | CK5^†^ | **EGFR**^†^ | **p63**^†^ |
| --- | --- | --- | --- | --- | --- | --- |
| TN1 | 28 | 2C | 3B | 1A | 4B | - |
| TN2 | 50 | - | 3C | 1B | 4C | 1B |
| TN3 | 38 | - | - | - | - | 2C |
| TN4 | 42 | 4C | - | - | 1B | - |
| TN5 | 60 | - | - | 4C | - | 3B |
| TN6 | 70 | - | 4C | 1A | - | 1B |
| TN7 | 69 | - | 4C | 2B | - | - |
| TN8 | 48 | - | - | - | 2A | - |
| TN10 | 71 | - | - | - | 1A | 1A |
| TN11 | 65 | - | - | - | - | - |
| TN12 | 34 | - | - | - | 3A | - |
| TN13 | 66 | - | - | 2A | - | 1B |
| TN14 | 56 | - | - | - | - | - |
| TN15 | 76 | 2B | - | - | - | - |
| TN16 | 46 | - | - | - | - | - |
| TN17 | 55 | 3B | 1B | - | - | - |
| TN18 | 57 | - | - | 1A | - | - |
| TN19 | 38 | - | 1C | 1C | 4A | 1A |
| TN20 | 57 | - | 3C | 4B | 4A | 4B |
| TN21 | 63 | - | 1C | 4C | 3B | 2A |
| TN22 | 56 | - | - | - | 4A | - |
| TN23 | 66 | - | - | - | 3B | - |
| TN24 | 67 | - | - | - | - | - |
| TN25 | 45 | - | 4C | 1A | 2A | - |
| TN26 | 41 | - | 4C | 4C | 4C | 4C |
| TN27 | 54 | - | - | - | 4C | - |
| TN28 | 57 | 3C | - | 3C | 3C | 2B |
| TN29 | 63 | 4B | - | - | - | - |
| TN31 | 59 | - | 2C | nd | - | - |
| TN32 | 46 | 2A | 1C | 4C | 3B | - |
| TN33 | 57 | - | - | 2A | 3B | - |
| TN34 | 59 | - | - | nd | - | nd |
| TN35 | 38 | - | - | 1A | 1C | - |
| TN36 | 55 | 4B | - | - | 4B | 2B |
| TN37 | 58 | - | - | - | - | - |
| TN38 | 77 | - | 4C | 3B | 1A | - |
| TN39 | 45 | 4B | 1C | 1A | - | 2A |
| TN40 | 68 | 4C | - | 2A | 1A | 2A |
| TN41 | 37 | - | 1C | 1A | 1A | - |
| TN42 | 52 | - | - | 4C | 4C | - |
| TN43 | 48 | - | 0 | - | 3B | - |
| TN44 | 33 | - | 1C | - | 3B | - |

* Age at diagnosis. ^†^ Expression of ESO, CK5, CK14, EGFR (epithelial growth factor receptor) and p63 was assessed by IHC staining of paraffin-embedded tumor tissue. The percentage of positive cells (-, 0-rare; 1, <10%; 2, 10-25%; 3, 25-50%; 4, >50%) and staining intensity (A, faint; B, moderate; C, strong) are reported. nd, not done.
